# The Role of Connexin Hemichannels in Inflammatory Diseases

**DOI:** 10.3390/biology11020237

**Published:** 2022-02-02

**Authors:** Bo Peng, Chengping Xu, Shuaiwei Wang, Yijie Zhang, Wei Li

**Affiliations:** Sepsis Laboratory, Center for Translational Medicine, Henan University, Kaifeng 475000, China; pb0729@126.com (B.P.); CP13125095726@163.com (C.X.); shuaiwei0909@163.com (S.W.)

**Keywords:** gap junctions, connexin hemichannel, mimetic peptide, innate immune cells, sepsis, ischemia, channel blocker, inflammation, ATP, HMGB1

## Abstract

**Simple Summary:**

Connexin hemichannels are formed by connexin protein family members, and connect cytosol and extracellular milium. The function of connexin hemichannels cannot be readily distinguished from the gap junctions formed also by connexins, or hemichannels formed by pannexins. It appears that connexin hemichannels are normally closed to maintain cellular homeostasis, but can be activated in pathophysiological processes to serve as toxic membrane pores. On the other hand, gap junctions are normally open in order to perform critical physiological functions, but are often closed or down-regulated under pathological conditions. The development and characterization of connexin mimetic peptides have resulted in a panel of connexin hemichannel-selective blockers. Investigations using these blockers have shown that the opening of connexin hemichannels facilitates the release of damage-associated molecular patterns, a class of endogenous molecules that are critical for the pathogenesis of inflammatory diseases. The blockade of connexin hemichannels virtually always leads to attenuated inflammation, reduced tissue injury and improved organ function. In this review, we provide an updated view of the role of connexin hemichannels in inflammatory diseases.

**Abstract:**

The connexin protein family consists of approximately 20 members, and is well recognized as the structural unit of the gap junction channels that perforate the plasma membranes of coupled cells and, thereby, mediate intercellular communication. Gap junctions are assembled by two preexisting hemichannels on the membranes of apposing cells. Non-junctional connexin hemichannels (CxHC) provide a conduit between the cell interior and the extracellular milieu, and are believed to be in a protectively closed state under physiological conditions. The development and characterization of the peptide mimetics of the amino acid sequences of connexins have resulted in the development of a panel of blockers with a higher selectivity for CxHC, which have become important tools for defining the role of CxHC in various biological processes. It is increasingly clear that CxHC can be induced to open by pathogen-associated molecular patterns. The opening of CxHC facilitates the release of damage-associated molecular patterns, a class of endogenous molecules that are critical for the pathogenesis of inflammatory diseases. The blockade of CxHC leads to attenuated inflammation, reduced tissue injury and improved organ function in human and animal models of about thirty inflammatory diseases and disorders. These findings demonstrate that CxHC may contribute to the intensification of inflammation, and serve as a common target in the treatments of various inflammatory diseases. In this review, we provide an update on the progress in the understanding of CxHC, with a focus on the role of these channels in inflammatory diseases.

## 1. Introduction

**Figure 1:**
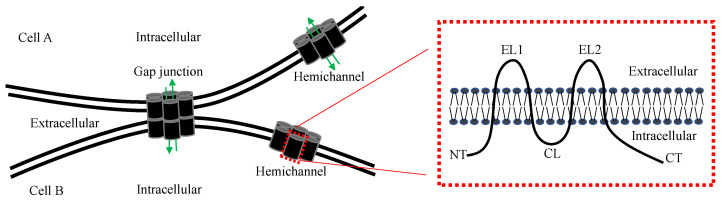
Schematic diagram of a gap junction, hemichannel and connexin. The membrane topology of a connexin protein is shown on the right. NT, N-terminal; EL, extracellular loop; CL, cytoplasmic loop; CT, carboxyl terminal. Green arrows indicate the gap junction and hemichannel pores that allow the passage of small molecules.

Connexins are a family of transmembrane proteins encoded by 21 connexin genes in humans and 20 in mice [1,2]. Connexin genes are designated with GJ, the abbreviation for gap junction, followed by Greek letters (e.g., GJα1), whereas connexin proteins are often identified by the predicted molecular mass in kilodalton (e.g., connexin43, Cx43) [1,2]. All connexins share the same topological structure, including four transmembrane domains (Figure 1), two extracellular loops (EL1 and EL2), a cytoplasmic loop (CL) and amino (NT) and carboxyl termini (CT) in the cytoplasm [3,4]. The CTs exhibit the greatest heterogeneity in amino acid sequences, whereas the transmembrane and extracellular domains are most conserved among the connexin isoforms [3,4].

In general, connexins are expressed in the endoplasmic reticulum, transported to the Golgi apparatus to form hexamers (connexons), trafficked to the plasma membrane as connexin hemichannels (CxHC), which then flow laterally to fuse with gap junction plaque and assemble with the CxHC from apposing cells, forming into gap junctions [2,4]. Therefore, CxHC exist on plasma membranes before forming gap junctions. Most connexins have a half-life of 1–2.5 h and, accordingly, CxHC are constantly replenished on plasma membranes [2]. The presence of CxHC does not always lead to the formation of gap junctions [5,6,7]. For example, hemichannels formed by Cx43, the most widely expressed connexin, can remain non-junctional on cell membranes if adequate adhesion molecules are not expressed [5]. Non-junctional connexin structures are also reported in vivo [8,9,10]. In a study using atomic force microscopy, hemichannels were found to account for about 12–17% of the total number of plaques in gap junction preparations from rat hearts [10], suggesting that CxHC may possess substantial permeation capacity in some critical organs.

The pore of CxHC has a maximal radius of approximately 14 Å, which allows the passive passage of small molecules (<1.5 kDa), including ions, nutrients, metabolites and signaling factors, such as IP3 and ATP [2,4]. By connecting the cytosol of coupled cells, gap junctions serve as intercellular communication channels. CxHC, on the other hand, provide a conduit between the cytosol and extracellular milieu [2,4]. CxHC are in a closed or low conductance state under physiological conditions to maintain intracellular homeostasis [11,12]. Indeed, the initial characterizations of functional CxHC showed that increased hemichannel permeability, resulting from an overexpression of Cx43 in macrophages and the ectopic expression of Cx46 in Xenopus oocytes, was associated with cell death [13,14]. In the last two decades, it has increasingly been recognized that CxHC opening may occur under physiological conditions, such as sheer stress, and is a frequent cellular response to various pathological conditions, such as obesity and various inflammatory diseases [4,12,15,16,17]. 

In this review, we will discuss recent developments in the methodology of CxHC research, the response of CxHC to microbiologically induced and sterile inflammation and the contribution of CxHC to tissue injury in inflammatory diseases. In addition, we will also briefly discuss some critical issues in CxHC research. 

## 2. Assessment of Hemichannel Permeability

CxHC capacity is determined by two factors, hemichannel quantity and permeability. The presence and quantity of CxHC on the cell membrane can be determined electrophysiologically, biochemically, immunohistochemically and microscopically [5,10,18,19,20,21]. The permeability of CxHC to charged permeants can be assessed via channel conductance, which has shown that CxHC formed by different isoforms have distinct electrophysiological characteristics [12,18,22]. In contrast to single channel permeability (or conductance), the overall hemichannel transport capacity of a cell or tissue is often determined by the uptake (extracellular application) or release (intracellular application) of fluorescent reporter molecules (e.g., Lucifer yellow) and bioactive molecules, such as ATP [12,19,23,24]. Bioactive molecules are preferred, as they are biologically relevant, but this approach is limited by its poor feasibility [12,25]. Fluorescent reporters are routinely used, especially in in vivo studies, due to advantages in availability, versatility and sensitivity [12,23,24]. However, these molecules can also pass through hemichannels formed by another protein family, pannexins [1,23,26,27]. In addition, membrane pores formed by gasdermin family members in pyroptotic cells are also permeable to reporter molecules, such as propidium iodide [28]. Therefore, CxHC-specific blockers should be employed to demonstrate the involvement of CxHC in permeability assays using reporter molecules.

## 3. Selective Manipulation of CxHC

Changes in connexin expression and mutations in connexin genes have been associated with various developmental defects, as well as non-hereditary diseases, demonstrating the functional significance of connexin channels [29]. However, efforts to differentiate the functions of gap junctions and CxHC have been met with challenges, largely arising from the poor selectivity of the interventions used to manipulate CxHC or gap junctions [4,23,30]. Because CxHC are formed prior to being assembled into gap junctions, all interventions interrupting connexin expression, trafficking and oligomerization would have similar effects on the formation of both channels [2,5]. Hemichannel reporters can pass through CxHC as well as PxHC, which often co-habit the same cell [12,23,30]. Thus, changes in hemichannel permeability, as indicated by these reporters, are not readily attributable specifically to CxHC or PxHC if no selective inhibitors are available. Moreover, virtually all pharmacological blockers are non-selective of gap junctions, CxHC or PxHT, and have collateral effects, defined and undefined, on the biological processes unassociated with these channels [23,30].

Given the disadvantages of the genetic and pharmacological manipulations of connexin channels, recent efforts in the development of CxHC blockers have focused on the heterogeneity in the amino acid sequences of connexins and the ready access to the extracellular domains of connexins in hemichannels [3,4,16]. These efforts have resulted in an array of CxHC blockers, including connexin-specific mimetic peptides and anti-EL antibodies [16,20]. The utilizations of these CxHC blockers have helped frame our understanding of CxHC functions, even though the limitations and underlying mechanisms of these CxHC blockers are yet to be fully characterized [4,16,17,30,31].

### 3.1. Extracellular Connexin Domain Mimetic Peptides

The amino acid sequences of ELs are highly conserved among connexin isoforms. Each of the two ELs is characterized by three cysteine residues that are essential for docking to the opposing CxHC to form gap junctions [1,3,16]. The ELs in non-junctional CxHC are exposed in extracellular space, but are largely inaccessible in gap junctions, due to the narrowness of the gap (40 Å) between the junctional membranes [2,4,16]. Such configuration renders the ELs of non-junctional CxHC readily accessible as substrates of extracellular elements.

Hemichannels formed by Cx43, the ubiquitously expressed connexin isoform, have been extensively investigated. The amino acid sequences of Cx43 ELs have been frequently used as templates for the design of mimetic peptide blockers. Significantly, 43Gap26 (VCYDKSFPISHVR) and 43Gap27 (SRPTEKTIFII) are among the first peptide mimetics of Cx43 ELs [32,33]. These peptides were initially shown to inhibit gap junctional intercellular communication (GJIC) by reducing gap junction formation presumptively through their complementary interaction with the ELs, thereby preventing the docking of opposing Cx43HC [4,12,16,32,33]. The blocking effects of 43Gap26 and 43Gap27 on Cx43HC have been tested in a broad variety of cells and were shown to occur within minutes after administration, suggesting that existing CxHC are indeed targeted by these peptide blockers [34,35]. Given the rapid turnover rate of Cx43 gap junctions, it is intriguing that the inhibitory effect of 43Gap26 and 43Gap27 can last for up to 6 h without significantly affecting GJIC [36]. On the other hand, the applicability of these peptides in CxHC studies is limited by such a time frame. Similar to 43Gap27, peptides 32Gap27 and 40Gap27, consisting of partial sequences of the EL2 of Cx32 and Cx40, can also block Cx40HC and Cx32HC, respectively [12,16,23]. In addition, Peptide5 (VDCFLSRPTEKT) was developed via the 5-amino acid N-terminal shift of 43Gap27. This peptide is more effective in blocking Cx43HC than its parental peptide, and exhibits a higher selectivity, as it does not affect GJIC when used at less than 100 μM [12,37].

However, not all such attempts are successful. For example, SRPTEKT-Hdc was created through the carboxylic lipidation of a peptide containing a sequence common to 43Gap27 and Peptide5. This peptide was initially shown to be five times more potent at inhibiting Ca^2+^ wave propagation and reporter dye uptake than 43Gap27, while it had little impact on GJIC during an application lasting 61–75 min [38]. Further investigations conducted by the same researchers showed that SRPTEKT-Hdc can block Cx43 gap junctions as well as hemichannels, especially when the S368 of Cx43 is phosphorylated [39]. 

In order to create more potent, CxHC-selective blockers, we recently screened a panel of hexamer peptides containing consecutive fractions of 43Gap26 [19]. Among these peptides, P5 (ENVCYD) showed a higher potency for suppressing Lucifer yellow uptake than the parental peptide (43Gap26) in macrophages, in which Cx43 is the predominant connexin isoform [19,40]. Importantly, a prolonged incubation of P5 (over 16 hr) did not cause a significant change in the expression and phosphorylation of Cx43 or intercellular dye transfer in NIH3T3 fibroblasts, suggesting that P5 can block Cx43HC, but not gap junctions [19]. Subsequently, we found that P5 can also reduce hemichannel permeability in human pulmonary microvascular endothelial cells, HepG2 cells and Cx43-deficient mouse cortical astrocytes [24]. In addition, P5 indiscriminately decreased hemichannel permeability in all LPS-treated alveolar cells in vivo [24]. These observations suggest that P5 is possibly a pan-CxHC inhibitor, perhaps due to the fact that the full sequence of P5 is present in the EL1 of Cx43, Cx33, Cx45, Cx46 and Cx50, and the sequence of the last five amino acids also exists in Cx26, Cx30, Cx30.3, Cx31, Cx31.1, Cx37, Cx39, Cx40 and Cx47 [1,2,24].

### 3.2. Intracellular Connexin Domain Mimetic Peptides

The amino acid sequences of intracellular domains exhibit a higher level of heterogeneity among connexin isoforms, and are therefore often the focus in the development of isoform-specific CxHC blockers [2,3,4,16]. A peptide mimetic of Cx43 CL, termed L2 peptide (DGANVDMHLKQIEIKKFKYGIEEHGK), was designed to block the interaction between the CL and CT of Cx43 [41]. The delivery of L2 to the cytosol, either directly by a whole-cell recording pipette or indirectly with the aid of a TAT tag, caused a reduction in CxHC-related membrane currents, dye uptake and ATP release [37,41,42]. Such effects can be reversed by a co-application of the peptide (CT10) mimetic of the last 10 amino acids (SRPRPDDLEI) in the CT. Similar to CT10, a shorter C-terminal peptide (CT9, RPRPDDLEI) and a peptide mimetic of the SH3-binding domain of Cx43 (SSPTAPLSPMSPPG) was also shown to prevent Cx43HC closure, likely by interfering with the CL–CT interaction [43]. These observations suggest a critical role for CL–CT interactions in the regulation of Cx43HC [41]. Interestingly, L2 keeps Cx43 gap junctions in a high-conductance state, demonstrating the dual-sided effects of L2 on gap junctions and hemichannels [37,41,42]. Gap19 (KQIEIKKFK) is a peptide mimetic of a CL sequence shorter than the L2 [44]. The Cx43HC-blocking property of Gap19 has been demonstrated in many cells and tissues [4,12,16,37,44]. Importantly, this peptide does not inhibit the gap junctions and hemichannels formed by pannexin1 or other connexins, suggesting that Gap19 is highly selective for Cx43HC [12,44]. Surprisingly, Gap19 was recently shown to markedly increase the uptake of Lucifer yellow in LPS-challenged macrophages and HepG2 cells [19]. Since LPS is known to induce cell injury, it remains to be clarified whether the Gap19-induced dye uptake was due to increased cell injury or elevated hemichannel permeability [19]. Similar to Gap19, 32Gap24 (YGRKKRRQRRRGHGDPLHLEEVKC) is a peptide mimetic of the CL of Cx32, and has been shown to block Cx32HC, but not gap junctions [45,46]. 

The inhibition of CxHC can also be achieved by reducing channel quantity. α–Connexin carboxyl-terminal peptide (ACT1) is a combination of a short sequence at the Cx43 CT (RPRPDDLEI) and an N-terminal antennapedia internalization sequence (RQPKIWFPNRRKPWKK). ACT1 can induce a significant increase in the size of gap junction plaques, while reducing the density of Cx43 immunoreactivity, indicating that ACT1 may facilitate the assembly of Cx43 gap junctions and, consequently, reduce the quantity of Cx43HC [47,48].

### 3.3. Extracellular Domain Antibodies

The exposure of connexin ELs in hemichannels makes these domains accessible to not only mimetic peptides, but also EL-specific antibodies [3,20,23]. Indeed, a number of EL antibodies have been generated and shown to be effective in blocking hemichannel permeability [20,49]. On the other hand, antibodies are much larger molecules (e.g., 150 kDa for IgG) than mimetic peptides (0.7–3 kDa). The binding of antibodies to ELs likely creates a hindrance to gap junction formation, which leads to a decreased GJIC [20,49]. Moreover, the cross-binding of EL antibodies to other connexin isoforms has also been observed, likely due to the well-conserved nature of ELs [20,23,49]. These limitations have largely restricted the application of these antibodies, especially when prolonged administration is required.

## 4. Association of CxHC with Pathogenesis of Inflammatory Diseases

### 4.1. Inflammatory Diseases

Inflammation is a defense mechanism against the deleterious effects of endogenous and exogenous pathogens [50,51]. Exogenous pathogens are microorganisms that have breached the epithelium. Infectious microorganisms and their degradation products, such as lipopolysaccharide (LPS), are called pathogen-associated molecular patterns (PAMPs), whereas injurious elements released from stressed or injured host cells are collectively categorized as damage-associated molecular patterns (DAMPs) [50,51]. PAMPs and DAMPs bind to pattern recognition receptors (PRRs), such as Toll-like receptors (TLRs) and NOD-like receptors (NLRs), and consequently trigger microbiologically induced and sterile inflammation, respectively [19,50,51,52]. The cellular inflammatory responses are predominantly carried out by innate immune cells, including monocytes/macrophages, neutrophils, dendritic cells and dormant macrophage-like cells, such as the Kupffer cells in liver and the microglia in the central nervous system (CNS) [52]. Some structural cells, such as endothelial, epithelial and astroglial cells, also contribute to organ-specific immune responses [52,53]. Upon the binding of PAMPs and/or DAMPs to PRRs, innate immune cells migrate to the site of infection and inflammation to clear PAMPs and DAMPs, while activating the adaptive immune system and increasing the expression and secretion of cytokines and chemokines. Proinflammatory cytokines, such as TNF-α and IFN-γ, and chemokines further augment the recruitment of inflammatory cells, which ultimately leads to the clearance of PAMPs and DAMPs, and a recovery from the infection or inflammation [50,52]. However, the prolonged presence of PAMPs and the sustained accumulation of DAMPs can cause excessive inflammation, which results in additional (secondary) tissue damages and contributes to the pathogenesis of various inflammatory diseases [50,54,55]. A growing body of evidence strongly suggests that CxHC intensify inflammation by facilitating DAMP release. 

### 4.2. Responses of CxHC in Innate Immune Cells to Inflammation

The transport capacity of CxHC is associated with the number of hemichannels, which is indicated by the level of connexin expression. The expression of connexin isoforms is cell- and tissue-dependent, except that Cx43 is expressed in virtually all tissues [2,16,56]. Consistently, Cx43 is found to be expressed by all innate immune cells, especially when these cells are activated [40,56,57,58]. Besides Cx43, monocytes, macrophages and neutrophils also express Cx37, whereas dendritic cells may also express Cx45. There is evidence suggesting that Kupffer and microglial cells may also express Cx26, and several other connexins, at least in cultured conditions [57,58,59]. While it is still controversial whether gap junctions are formed between innate immune cells, such as macrophages [57,58,59,60], heterocellular gap junctions between innate immune cells and other cell types, such as epithelial and endothelial cells, are likely present, and may perform important functions [61,62,63,64]. 

The Gram-negative bacterial endotoxin, LPS, is the most commonly used PAMP in studies of the inflammatory responses of innate immune cells, and has been shown to stimulate Cx43 expression in the majority of innate immune cells [56,57,58,59,60]. Similar to LPS, peptidoglycan, a PAMP highly expressed in Gram-positive bacteria, also shows a stimulatory effect on Cx43 expression in microglia [65]. In contrast to PAMPs, proinflammatory cytokines, such as TNF-α, IL-6, IL-1β or IFN-γ, often need to be applied in combination with another cytokine or LPS to elicit a significant effect on connexin expression in most studies [56,57]. On the other hand, an individual cytokine may trigger a redistribution of CxHC on the cell membrane, as suggested by the increased formation of heterocellular gap junctions between innate immune cells and other cells [16,58,61,62,63,64]. These observations indicate that connexin expression is likely regulated by the downstream signaling of PRRs, but not cytokine receptors. Indeed, we have previously found that an LPS-induced increase in Cx43 expression is completely abolished in TLR4-, but not RAGE- or TLR2-deficient primary peritoneal macrophages [19]. The TLR-dependent regulation of connexin expression is not limited to PAMPs. Serum amyloid A (SAA), an acute-phase proinflammatory protein, showed a similar potency to LPS in the stimulation of Cx43 expression in macrophages. Interestingly, the stimulatory effect of SAA is also mediated by TLR4 [19]. 

The impact of DAMPs on connexin expression is more divergent than PAMPs (Table 1). For instance, an increase in the expression of connexins, such as Cx26, Cx32 and Cx43, can be induced by the application of DAMPs, such as biglycan, versican, hyaluronan, heparan, fibronectin and high-mobility group box-1 (HMGB1), in fibroblast, astrocytes and alveolar epithelial cells [66,67,68,69,70,71,72]. Conversely, other DAMPs, such as uric acid and heat-shock proteins, can suppress the expression of Cx26 and Cx43 in astrocytes, cardiac myocytes, vascular endothelial and colorectal cancer cells [73,74]. Somewhat surprisingly, the effects of DAMPs on connexin expression in innate immune cells have received less attention. HMGB1 is perhaps the most widely investigated proinflammatory DAMP [75]. In contrast to the potent stimulatory effects of LPS and recombinant SAA, a parallel application of recombinant HMGB1 only caused a very mild change in Cx43 expression in macrophages [19], which is intriguing, given that HMGB1 is also a stimulatory ligand of PPRs, such as RAGE and TLR4 [75]. On the other hand, HMGB1 appears to increase Cx43 expression in astrocytes [72], indicating that a PRR-independent mechanism might be involved in the regulation of Cx43 expression. Indeed, it was shown that HIV-tat, the transactivator of HIV, can up-regulate Cx43 expression in these cells by directly binding to the Cx43 promoter [76]. Nevertheless, these results suggest that PAMPs appear to be more consistent stimulators of connexin expression than the DAMPs in innate immune cells. 

Given that few gap junctions are formed between innate immune cells, the PAMP-induced up-regulation of connexin expression in these cells likely causes an increase in CxHC transport capacity [19,57,60]. Consistently, the administration of PAMPs virtually always results in an increase in hemichannel activity in innate immune cells [19,56,57,78], suggesting the possibility that CxHC may mediate PAMP-induced inflammatory responses.

### 4.3. CxHC in Pathogenesis of Inflammatory Diseases

Differences in the cellular compartments connected by gap junctions and hemichannels dictate that these connexin channels perform distinct functions. Accumulating evidence suggests that gap junctions and CxHC play an opposite role in the pathogenesis of inflammatory diseases [16,17,30]. In particular, gap junctions help maintain intracellular homeostasis, and are therefore considered to be “good channels”, whereas CxHC may largely serve as “bad channels”, as the opening of CxHC leads to “leaky” membranes, cell injury, the extracellular accumulation of DAMPs and, ultimately, secondary inflammatory tissue damage [17]. 

With the understanding that genetic and pharmacological manipulations are poorly selective for CxHC, the majority of recent investigations of CxHC in inflammatory diseases have employed peptide blockers [4,16,17,19,24,30]. In over twenty-six inflammatory diseases and disorders, the blockade of CxHC has invariably led to reduced tissue injury and improved organ function (Table 2). In most cases, CxHC inhibition is associated with attenuated inflammatory responses, including decreased cytokine levels or the suppressed recruitment of inflammatory cells [19,24,79,80,81,82,83,84,85,86,87,88,89,90,91,92,93,94,95,96,97,98,99,100,101,102,103,104,105,106,107], suggesting that the attenuation of inflammation is likely a mechanism underlying the protective effect of CxHC inhibition. Because of the similarities in experimental methodology and the observed effects of these studies, we choose sepsis and ischemia as examples to illustrate the role of CxHC in inflammatory diseases involving microbiologically induced and sterile inflammation, respectively. 

#### 4.3.1. Sepsis, a Microbiologically Induced Inflammatory Disease 

Sepsis is a life-threatening organ dysfunction syndrome caused by host responses to an infection, and is related to 11 million deaths annually worldwide [109,110]. While the nature of these host responses is yet to be fully defined, numerous studies suggest that dysregulated systemic inflammation is critical for tissue injury and consequent organ dysfunction [111,112]. The involvement of CxHC in sepsis pathogenesis was first implicated when we examined the effect of carbenoxolone, a common gap junction and hemichannel blocker, on sepsis lethality in a mouse model of polymicrobial sepsis. Carbenoxolone increased the survival rate when used at lower doses (5–10 μmol/kg), whereas higher doses of carbenoxolone (50–100 μmol/kg) led to the increased mortality of septic mice [113]. In a following study, it was found that both Gap26 and P5 were protective in septic mice, suggesting that the opening of CxHC contributes to sepsis-related organ injury [19]. Consistent with our findings, Delvaeye et al. found that blocking Cx43HC with Gap19 also exhibited protective effects in a mouse model of inflammatory shock induced by TNF, whereas enhancing Cx43HC with CT9 showed opposite effects [105]. More recently, Dosch et al. investigated the role of Cx43 channels in sepsis using Lyz2cre/cre*GJ*α1flox/flox mice, in which the Cx43 gene was specifically deleted in macrophages. Compared with wild-type counterparts, these mice showed a higher survival rate, in addition to reduced cytokine levels [104]. These observations demonstrate that the Cx43HC in macrophages likely contribute to sepsis pathogenesis, at least in the animal model of sepsis.

Acute respiratory distress syndrome (ARDS) is a severe respiratory failure, and is most commonly caused by sepsis [114,115]. The etiology of ARDS is routinely investigated using models of acute lung injury [115]. Similar to other organs, Cx43 is the most abundantly expressed connexin, among at least seven connexin isoforms (i.e., Cx26, Cx31, Cx32, Cx37, Cx40, Cx43, Cx45 and Cx46) [116,117]. In an early study of the role of Cx43 in acute lung injury, a 45 min incubation of 43Gap26 and 43Gap27, a treatment believed to close hemichannels but not gap junctions, completely prevented the thrombin-induced microvascular permeability, probably by inhibiting Cx43HC in alveolar endothelial cells [118]. Consistently, we recently found that P5, the broad-spectrum CxHC blocker, reduced hemichannel permeability, alveolar neutrophil infiltration and mortality in an acute lung injury model induced by intratracheal LPS instillation [24]. These observations suggest that CxHC play an important role in pulmonary inflammation and injury. In contrast to CxHC, gap junctions may be protective in lung injury. For example, Westphalen et al. found that residential alveolar macrophages can form Cx43 gap junctions with epithelial cells, but do not exhibit any hemichannel activities [64]. The specific deletion of the Cx43 gene in these macrophages, but not bone marrow-derived macrophages, resulted in a higher level of cytokines/chemokines in bronchoalveolar lavage fluid and higher mortality in a mouse model of LPS-induced lung injury, suggesting that the GJIC between sessile alveolar macrophages and epithelial cells is protective [64].

#### 4.3.2. Ischemic Tissue Injury, a Sterile Inflammatory Disease

Tissue injuries caused by ischemia or ischemia–reperfusion (IR) can be separated into two phases: the first is caused directly by hypoxia and hypoglycemia during blood vessel occlusion, and the second is attributable to the ensuing inflammation [119,120]. DAMPs released from injured cells in the first phase play a major part in triggering and intensifying inflammation in the vicinity of ischemic loci [120]. Because such inflammation is not caused by infectious pathogens, it is classified as sterile inflammation [50,120]. In addition to ischemic injury, the pathologies of many other diseases, such as atherosclerosis, Parkinson’s disease and Alzheimer’s disease, are also associated with inflammatory cell damages [121]. 

The role of CxHC has been investigated in the ischemic injury of several organs, including the brain, heart, liver and retina [19,99,108,122,123,124]. The brain parenchyma is primarily composed of neurons and glia, and the latter is subcategorized into astrocytes, microglia and oligodendrocytes. Ischemia in the brain can be divided into three regions: the infarct core, containing the majority of dead neurons; the infarct-surrounding penumbra, where neurons are endangered and salvageable; and the healthy area in the periphery of the penumbra [125,126]. Cx36 and Cx45 are the major neuronal connexins, and form gap junctions as well as functional hemichannels [56,127,128]. Although it has been shown that the level of Cx36 expression correlates with the degree of excitotoxic or ischemic neuronal death, the role of Cx36HC is not yet clear [127]. Moreover, there has been no study of neuronal Cx45HC. Microglia are resident macrophages in the CNS. Cx43 is not detected in inactive (resting) microglia, but has been reported in activated microglial cells [57]. There has been no evidence of the role of microglial CxHC in CNS inflammation. On the other hand, the proinflammatory cytokines IL-1β and TNF-α secreted by microglia can increase Cx43HC activity in astrocytes [129].

Astrocytes express a high level of Cx43, in addition to Cx26 and Cx30 [130]. Under physiological conditions, astrocytes are well connected by gap junctions to form a so-called “functional syncytium”, maintaining CNS homeostasis by facilitating the dissipation of potentially harmful substances [125,130]. The permeability of astrocytic Cx43HC is elevated during ischemia and metabolic inhibition [131]. The inhibition of Cx43HC permeability by administering 43Gap26, GAP19 or Peptide5, or by reducing Cx43 phosphorylation, have all resulted in reduced cerebral IR-induced neuronal injury [98,132,133,134]. Interestingly, Peptide5 administration before and during cerebral ischemia did not cause any significant changes in the EEG, whereas post-ischemia administration resulted in an enhanced recovery, indicating that the protective effect of Peptide5 resides in the prevention of the secondary injury [120,132]. Notably, the neuroprotective effect of Peptide5 appeared to be limited to lower concentrations (50–100 uM) that are effective at blocking Cx43HC, since a higher concentration of Peptide5 (500 uM), which can also block GJIC, actually increased ischemic injury [134], suggesting an opposite role of Cx43 GJIC and Cx43HC. Similar to the neuroprotective effect of CxHC blockers in cerebral ischemia, both the administration of 43Gap27 and the astrocyte-specific deletion of the Cx43 gene conferred protection on ganglion cells after retinal ischemia–reperfusion [99,135].

It is noteworthy that the ischemic penumbra is characterized by the massive dephosphorylation of astrocytic Cx43 [125]. Given the association between Cx43 dephosphorylation and the increased permeability of astrocytic Cx43HC [131,136], the unique localization of dephosphorylated Cx43 indicates that astrocytic Cx43HC in the penumbra are perhaps the preferential (or more concentrated) targets of CxHC blockers. In this area, the blockade of astrocytic Cx43HC in the penumbra may provide more direct benefits to adjacent endangered neurons. 

Cx43, as well as CxHC, forms gap junctions in the heart. Gap junctions are predominantly located in the intercalated discs between cardiac myocytes, and serve to facilitate the propagation of electrical excitation, whereas CxHC are mainly located in an area (termed perinexus) surrounding the intercalated discs [21,137]. Similar to the CNS, cardiac CxHC are also induced to open during ischemia/hypoxia [138,139]. The inhibition of CxHC with 43Gap26, GAP19 or Peptide5 reduces ischemia-induced heart damage [21,44,96,108,123,140]. In contrast to Cx43HC blockers, a PxHC blocker (i.e., 10Panx1) did not show any protection [44,123]. Taken together, these studies demonstrate that Cx43HC, probably not PxHC, contribute to further cardiac injury in ischemia. 

Besides connexin mimetic peptides, the applications of other CxHC blockers have also shown that CxHC are detrimental in inflammatory diseases. For example, the administration of tonabersat, an anti-migraine drug and a Cx43HC blocker, appears to be beneficial in diabetic retinopathy and age-related macular degeneration [141,142]. Similar to the deleterious effect of Cx43HC in many diseases, the hemichannels formed by Cx37 and Cx43 may contribute to the development of atherosclerosis [63,143]. 

### 4.4. The Control of DAMP Release by CxHC

It is important to note that the tissue protection conferred by CxHC blockers is always accompanied by attenuated inflammation in studies where the levels of cytokines or leukocyte recruitment were examined. Give the critical role of DAMPs in inflammation and tissue injury, these observations suggest that CxHC may mediate the release of DAMPs. Surprisingly, the relationship between CxHC and DAMP release has only been studied with four of the 30 recognized DAMPs [144].

#### 4.4.1. Mediation of ATP Release by CxHC

ATP is the energy-providing molecule essential for cell metabolism. The level of extracellular ATP (eATP) is very low under physiological conditions, and ATP serves as a signaling molecule in a variety of biological activities. During inflammation, however, the concentration of eATP can rise several hundred folds, and it functions as a DAMP by binding to purinergic receptors [145]. For instance, eATP can trigger inflammasome activation, neutrophil mobilization and T-cell suppression, and thereby causes inflammatory tissue injury [146]. The accumulation of eATP results from the passive release of dying or dead cells, and active release through Ca^2+^-dependent exocytosis or from channel pores on the cell membrane, such as CxHC and PxHC [55,147].

The association between the opening of CxHC and ATP release was observed over twenty years ago. In these early studies, the increase in eATP occurred after CxHC were stimulated to open by a reduction of extracellular Ca2^+^ or metabolic inhibition [131,148]. The availability of CxHC-selective blockers has enabled extensive studies, providing strong evidence of the presence of a causative relationship between CxHC opening and ATP release (Table 3). In innate immune cells, such as macrophages and neutrophils, LPS-induced ATP release was inhibited or completely abolished by the administration of 43Gap27 and P5, or the deletion of the Cx43 gene, but not by the PxHC blocker probenecid or the exocytosis inhibitor N-Ethylmaleimide [19,104,149]. ATP release by various non-immune cells can also be inhibited by hemichannel blockers, including Gap19, 43Gap26, 43Gap27 and Peptide5, even though these reagents have varied selectivity for Cx43HC [19,24,34,46,94,104,139,149,150,151,152,153,154,155,156]. In keratinocytes, a reduction in eATP was seen after blocking Cx30HC with an EL antibody [157]. In collecting duct cells, in which Cx30 forms hemichannels in the apical membrane, both the hemichannel activity and eATP level were reduced in Cx30-deficient cells [158]. Given that CxHC are permeable to ATP, these studies demonstrate that ATP is likely released through CxHC during inflammation, and thereby intensifies inflammasome activation [155].

#### 4.4.2. Mediation of HMGB1 Release by CxHC

HMGB1 is a ubiquitously expressed nuclear protein. Under physiological conditions, HMGB1 functions to stabilize the nucleosomal structure and regulates gene transcription. In diseases, however, HMGB1 can be released passively by injured and dying cells due to membrane destruction [55,75]. Indeed, HMGB1 release has been observed in association with virtually all types of cell injuries, such as necrosis, apoptosis, necroptosis, pyroptosis and ferroptosis [55]. On the other hand, HMGB1 can also be actively released by immune as well as non-immune cells [19,55,75]. Extracellular HMGB1 can act like a paracrine proinflammatory cytokine and chemokine to augment local inflammation, or serve as a mediator of systemic inflammation to contribute to inflammatory tissue injury [55,75].

The active release of HMGB1 involves three steps: translocation from the nucleus to the cytoplasm, cross-cytoplasm trafficking to the cell membrane and exocytosis. The translocation of HMGB1 from the nuclei to the cytoplasm begins with the dissociation of HMGB1 from the chromosomes, which is enabled by the JAK/STAT-1-mediated acetylation of the nuclear localization sequences within HMGB1 [159]. The dissociated HMGB1 is then transferred to the cytoplasm by HSP90AA1, a heat-shock protein, and XPO1, a nuclear export protein [160]. The subsequent cross-cytoplasm trafficking is facilitated by the phosphorylation of double-stranded RNA-activated protein kinase R (PKR) [161]. Because HMGB1 does not have a signal peptide, it is not yet clear how it is ferried across the cytoplasm or secreted from the cell. 

There is evidence supporting the possibility that HMGB1 may be secreted in exosomes or microvesicles in a manner regulated by Cx43HC. In an earlier study, we found that both the hemichannel permeability of LPS-challenged macrophages and the HMGB1 level in culture medium can be reduced by carbenoxolone, a non-selective connexin and pannexin channel blocker [113]. Surprisingly, a parallel analysis showed that the levels of most cytokines and chemokines, as evaluated with an antibody array, did not change significantly, suggesting a selective association between hemichannel permeability and HMGB1 release [113]. Subsequently, we found that P5, the CxHC blocker, also attenuated LPS-induced HMGB1 release, but did not alter the protein level and phosphorylation state of PKR [19], indicating that the mediation of HMGB1 release by CxHC occurs after HMGB1 is transported across the cytoplasm. Similar to macrophages, LPS-induced HMGB1 release from human endothelial cells can also be inhibited by P5 [24]. Interestingly, both HMGB1 and Cx43HC were found to exist in exosomes [162,163,164], indicating that Cx43HC may be part of the carrier that delivers HMGB1 across the plasma membrane. Coincidentally, we found in an earlier study that metabolic inhibition, which causes increased Cx43 dephosphorylation and hemichannel permeability, led to a dissociation of Cx43 from β-actin, a cytoskeleton protein [136]. Taken together, it may be reasonable to hypothesize that the opening of Cx43HC triggers the fusion of Cx43HC with HMGB1-containing vesicles at the plasma membrane, dislodges the Cx43HC-HMGB1 complex from the cytoskeleton and subsequently releases HMGB1 into the extracellular space.

Similar to ATP and HMGB1, CxHC permeability is also associated positively with the release of S100β by enteric glia [150], but negatively with tenascin C in gingival fibroblasts [94]. It remains to be determined whether and how CxHC is involved in the regulation of the release of other DAMPs. Interestingly, Cx43 has binding motifs for RNA and DNA, which indicates that Cx43 may serve as a molecular transporter or, alternatively, a regulator of gene expression [165]. 

It is well recognized that CxHC are assembled in Golgi apparatus. These channels have also been reported in mitochondria. Mitochondrial CxHC may participate in the regulation of cell injury [21]. On the other hand, CL- and CT-based peptides, such as Gap19, need to enter the cytosol to be effective. Small EL-based peptides, such as P5, may be able to enter the cell through open HC. Once in the cell, these CxHC blockers may interfere with the CxHC in these organelles. Therefore, further investigations may be needed to precisely define the targets of CxHC blockers and further clarify the mechanisms underlying the role of CxHC in inflammatory diseases.

## 5. Concluding Remarks

In sync with the development and characterization of CxHC blockers, the role of CxHC has been extensively studied. Although experimental data need to be interpreted with caution, a consensus is emerging that CxHC mediate DAMP release, thereby contributing to the pathogenesis of many inflammatory diseases. Hence, CxHC may be considered to be a common therapeutic target in the treatment of these diseases or disorders. It should be noted that PxHC not only share many molecular and physiological characteristics with CxHC, but also contribute to the pathogenesis of inflammatory diseases. For instance, PxHC promote inflammasome activation, viral replication and infection (e.g., HIV and SARS-CoV-2) [166,167,168]. A combined blockade of CxHC and PxHC may prove to be more effective in the treatment of some inflammatory diseases than targeting either type of hemichannels.

## 6. Patents

Author Wei Li is an inventor of P5-related patents in the US and China.

## Figures and Tables

**Table 1:** Effects of DAMPs on connexin expression.

DAMP	Connexin	Connexin Expression	Cell/Tissue	Specie	Reference
Biglycan	Cx32, Cx43	↑	Heart	Mouse	[69]
Versican	Cx43	↑	Fibroblasts	Human	[70]
LMW hyaluronan	Cx26, Cx32, Cx43	↑	Fibroblasts, astrocytes	Human	[67,71]
Heparan sulfate	Cx26	↑	Hepatocytes	Rat	[66]
Fibronectin	Cx43	↑	Type II alveolar epithelial cells	Rat	[68]
Uric acid	Cx43	↓	Myocardial cells	Human	[74]
Heat-shock proteins	Cx26, Cx43	↓	Vascular endothelial cells, colorectal cancer cells	Human	[73]
Aβ_25–35_	Cx43	↑	Astrocytes, microglia, neurons	Mouse	[77]
HMGB1	Cx43	↑	Astrocytes	Mouse	[72]

↑, increased expression; ↓, decreased expression.

**Table 2:** Effects of peptide connexin hemichannel blockers on human and animal models of inflammatory diseases.

Disease/Disorder	Species	Model	Blocker	Main Effect	Reference
Acute lung injury	Rat	LPS and HCl instillation	Gap27, P5	Reduced mortality, lung injury and leukocyte recruitment	[24,79]
Age-related macular degeneration	Rat	Intense light exposure, laser photocoagulation	P5, ACT1	Reduced inflammation, improved retinal pigment epithelium and function	[80,81,82]
Alzheimer’s disease	Mouse	APP_swe_/PS1_dE9_ mice	Gap26	Reduced gliotransmitter release	[83]
Cardiac injury	Mouse	Left ventricle cryoinjury	ACT1	Reduced inducible arrhythmia	[84]
Chronic diabetic foot ulcers	Human	Neuropathic foot ulcer in diabetic patients	ACT1	Improved ulcer re-epithelialization	[85]
Chronic venous leg ulcers	Human	Ulcer patients	ACT1	Faster ulcer closure	[86]
Chronic pain	Mouse	Chronic constriction injury; peripheral neuropathy	Peptide5	Reduced mechanical pain	[86]
Corneal epithelial wounding	Human, rat	Ex vivo human cornea, suture-induced corneal inflammation, corneal wounding by isopropyl alcohol	Gap27, ACT1	Improved healing and reduced inflammation	[88,89]
Diabetes, type I	Rat	Streptozotocin injection	ACT1	Improved wound closure and reduced inflammation	[90]
Diabetic retinopathy	Mouse	Intravitreal injection of IL-1b and TNF-a	Peptide5	Improved function and reduced inflammation and microglia infiltration	[91]
Duchenne muscular dystrophy (+ arrhythmias)	Mouse	Isoproterenol challenge in DMD mice	Gap26, Gap19	Decreased animal death and cardiac arrhythmogenesis	[92]
Fetal asphyxia	Sheep	Complete umbilical cord occlusion (25 min)	Peptide5	Reduced neuron and oligodendrocyte death	[93]
Gingival wound healing	Human	Gingival wound healing	Gap19	Faster wound healing	[94]
Intracerebral hemorrhage	Mouse	Collagenase IV injection	Gap19	Reduced cytokine levels and neurological deficits	[95]
Ischemia, heart	Rat, mouse	Cardiac ischemia/reperfusion	Gap26, Gap27, Gap19	Reduced infarct size	[43,96,108]
Ischemia, cerebral	Rat, sheep	Carotid artery occlusion and reperfusion	Gap19, Gap26, Gap27, Peptide5	Reduced cerebral infarct volume and neuron loss; improved functional recovery	[93,97,98]
Ischemia, hepatic	Mouse	Ischemia/reperfusion	P5	Reduced transaminases and LDH	[19]
Ischemia, retinal	Rat	Ischemia	Peptide5	Reduced vascular leakage and retinal ganglion cell loss	[99,100]
Liver fibrosis	Mouse	Thioacetamide	Gap19	Reduced fibrosis and inflammation	[101]
Parkinson’s disease	Mouse	1-methyl-4-phenyl-1,2,3,6 tetrahydropyridine-triggered dopamine neuron degeneration	Gap26, Gap19	Reduced dopamine neuron loss and microglial activation	[102]
Scarring	Human	Skin incision	ACT1	Less scarring, improved scar pigmentation	[103]
Sepsis	Mouse	Peritonitis	Gap27, P5	Reduced mortality	[19,104]
Septic shock	Mouse	TNF-induced septic shock	Gap19	Reduced mortality	[105]
Spinal cord injury	Rat	Mild contusion injury at T10	Peptide5	Improved motor neuron survival and hind limb function	[106,107]
Steatohepatitis, non-alcoholic	Mouse	Choline-deficient high-fat diet	Gap19	Reduced inflammatory markers	[46]

**Table 3:** Effects of connexin hemichannel blockers on DAMP release.

DAMP	CxHC Inhibition	Targeted Connexin	Extracellular DAMP	Cell/Tissue	Species	Reference
Tenascin C+	Gap19	Cx43	↑	Gingival fibroblasts	Human	[94]
S100 proteins	Fluoroscetate	Cx43	↓	Enteric glia	Mouse	[150]
ATP	P5, Gap27	Cx43	↓	Macrophages	Mouse	[19,104]
	Gap19, Gap26	Cx43	↓	Astrocytes	Mouse, rat	[77,151,152]
	Gap19, Gap27	Cx43	↓	Urothelial cells	Human, mouse	[153]
	Flufenamic acid, Gap26, Gap27	Cx43	↓	Endothelial cells	Bovine, human	[34,154]
	Gap26, 18a-glycyrrhetinic acid	Cx43	↓	Cardiac myocytes	Rat	[139]
	Gap27	Cx43	↓	Neutrophils	Human	[149]
	EL antibody	Cx30	↓	Keratinocytes	Human, mouse	[157]
	Gap19, Gap24	Cx32, Cx43	↓	Hepatocytes	Rat	[46]
	Peptide5, Gap24	Cx43, Cx32	↓	Epithelial cells	Human	[155,156]
	Cx30-null	Cx30	↓	Collecting duct cells	Mouse	[158]
HMGB1	P5	Cx43	↓	Macrophages	Mouse	[19]
	P5	Cx43	↓	Vascular endothelial cells, lung	Human, mouse	[24]

↑, increase; ↓, decrease.

## Data Availability

Not applicable.
